# Promoting healthier food purchases via social media: the role of polls and their visual features

**DOI:** 10.3389/fnut.2026.1726586

**Published:** 2026-04-10

**Authors:** Lars Bläuer, Sabine Maja Bremermann-Reiser, Lea Laasner Vogt, Ester Reijnen

**Affiliations:** School of Applied Psychology, Zurich University of Applied Sciences, Zurich, Switzerland

**Keywords:** descriptive social norms, healthy diet, nutrition, polls, purchase intention, social media

## Abstract

More and more food and beverage companies utilize *social media* to promote high-energy, low-nutrient products. The question is whether *descriptive* social norm feedback, implemented as a social media *poll*—in combination with its visual features and people's own (past) behavior—reduce the intention to purchase such advertised products. In Study 1, the participants' (*N* = 227) past behavior was evaluated based on their responses to the differently worded poll question: “Do you believe that your diet is (un)healthy?” (yes/no). Poll results were displayed proportionally (e.g., on the left—with a smaller bar—representing 27% yes answers), and participant's answer was marked with coloring the appropriate bar part in gray. Participants then rated their purchase intentions for healthy and unhealthy food products. Participants following a healthy diet showed significantly higher purchase intentions for healthy products, especially when the minority feedback of 27% appeared on the left side. No such effect appeared for participants with an unhealthy diet. In subsequent studies, visual features such as proportionality and color were systematically removed, causing the pattern of results to change or disappear. Overall, it appears that when using descriptive social norms feedback, its visual features should not be neglected.

## 1 Introduction

As billions of people around the world are now social media users (1), more and more companies are using social media platforms such as Instagram to market food products. Unfortunately, this marketing focuses on high-energy, low-nutrient foods^1^ contributing to *poor diets* [e.g., (2–5); see also (6)] and thus to the “obesity^2^ crisis” [e.g., (7, 8)]. In 2023 alone, around 40% of the adults in the USA were obese [(9); and this figure is predicted to rise; see (10)]. Furthermore, obesity is no longer limited to industrialized countries, but also affects less developed countries [e.g., (10)]. Considering this worldwide crisis, which is the fifth ranking cause of global mortality (11), successful counter-interventions are needed.

Interventions are needed that can—as a start—influence people's *intentions* to change. Most studies show that people's intentions (to perform a certain behavior) are also translated into (actual) behavior [see (12) for fruit and vegetable consumption]. This is based on the theory of planned behavior [e.g., (13, 14)]. However, there are also studies which show that the intention-behavior relationship is less clear-cut [intention-behavior gap research; see (15, 16)]. Some of these studies claim that the intention-behavior relationship is, for example, *moderated* [e.g., by customer satisfaction; (90)], while other studies claim that this relationship only exists in certain *subgroups*. Regarding this idea of subgroups, Sheeran (17) found that the intention-behavior relationship can only be observed in one group, namely people who want to change, but not in the other group, which he refers to as “inclined abstainers.” Regardless of the possibility of an intention-behavior gap or its (further) causes [i.e., see also the cold-to-hot empathy gap, (18)], the question arises as to which interventions could strengthen people's intentions to follow a healthier diet? Moreover, which of these interventions could possibly be implemented via social media?

One such possible intervention could be the simple provision of *health messages* (e.g., “Eating five servings of fruit and vegetables a day can improve your health [...]” from 38, p. 1060) highlighting the positive effects of a healthy diet. However, this intervention has proven to be of limited success in changing dietary choices (19–21). Presumably, this is because people dislike needing to make cognitive effort, or the so-called System 2 thinking [see (22)]. Humans are social beings and therefore often spontaneously adapt to others' behavior (through the use of System 1 thinking, which is effortless). Hence, leveraging social norms might be a more successful intervention. Social norms are defined as “rules and standards that are understood by members of a group, and that guide or constrain social behaviors without the force of law” (23, p. 152). The social behaviors referred to can cover all areas from sustainability and health to nutrition. The problem, however, is that people are not always able to correctly assess the actual behavior underlying a norm. Consequently, they mistakenly assume, for example, that alcohol consumption among students is high and/or widespread [so-called “normative misperceptions”; see, for example, (23)]. This could make alcohol consumption a desirable behavior and lead to students drinking more [see (24)]. Hence, the influence of these misconceptions on intention or behavior may be greater than the influence of the social norm itself [e.g., (25, 26), on the consumption of unhealthy snacks or (27), on the consumption of sugary drinks]. These misperceptions can, however, be counteracted by informative feedback (or appeals) about *actual* reported norms [e.g., (28, 29)]. This can be done in two ways. The first is by providing descriptive norm feedback^3^, which describes the *actual* behavior^4^ exhibited by a norm reference group in a given situation. Alternatively, injunctive norms feedback can be provided, which describes the behavior that *should* or *must* be exhibited within a norm reference group in a given situation (30–32). Research has shown that social norms feedback is quite effective [see (31–34, 55)^5^]. Descriptive norms are proven to be more effective than injunctive norms in promoting intentions/behaviors to follow a healthier diet. This is because they provide an immediate behavioral anchor, while the abstract appeals in injunctive norms appear to provoke reactance [(25, 35); see (36), and the use of dynamic norms to avoid this kind of reactance]. To illustrate the use of descriptive norms, we will take a closer look at the study by Claassen et al. (37). In their study, participants were randomly assigned to either a descriptive norm promoting fruit or vegetable consumption or no norm (control group) and were then asked to complete a survey (Time 1). A few days later (Time 2), participants were informed that some of their survey data had been lost and asked if they would participate in the study again. As compensation, they could choose between a fruit or vegetable basket. They then entered their choice into a pre-prepared (manipulated) Excel sheet. Depending on the descriptive norm condition they were assigned to (fruit or vegetable group), the Excel sheet contained more people who had chosen the corresponding basket (e.g., fruit basket in the fruit group). In the no norm or control group, an equal number of participants had chosen one of the two baskets. It was found that participants who were exposed to a vegetable norm were almost three times more likely to choose the *vegetable* basket (42%) than those who were exposed to a fruit norm (16%) or no norm [18%; see also (38–41)]. However, it is unclear why the descriptive norm worked for vegetables but not for fruit (one possible explanation is that of a ceiling effect). One should note, however, that there are also studies [e.g., (33)] which consider injunctive norms to be more effective than descriptive norms, or studies [e.g., (42)] that have found no difference between these two types of norms^6^. Nevertheless, it is worth considering that the study by Robinson et al. (38) showed that messages about social norms (especially descriptive ones) are more effective than health messages [see also (43) on environmental messages].

However, regarding the success of descriptive norms, the question arises as to whether their success depends significantly on whether the specified (actual or hypothetical) behavior is exhibited by a *majority* or a *minority* (reference group). Note that a reference to, for example, a majority group is established by using terms such as “most” or “the majority” [see, for example, (33), in the study on petrified wood; but see, Claassen et al. (37), who used an Excel sheet] or, more commonly, by using percentages (e.g., 74%). Research has shown that descriptive norms that refer to *majorities* (e.g., “Most students eat a lot of vegetables”) as a reference group are more successful [e.g., (44, 45)]. This is probably due to their function as social proof; [see, for example, (31, 46–48), Experiment 2, that is, indicating a behavior that has been proven to be effective for others]. In this context, however, it is less clear why referencing majorities is so successful. Is it due to how the norm feedback is phrased, positively [e.g., “(Most students) often eat vegetables”] or negatively [e.g., “(Most students) never eat meat”], that is, whether it promotes desired behavior or discourages undesired behavior? While Mollen et al. (49), for example, found that both formulations (positive/negative) promote norm-compliant behavior to the same extent, Cialdini et al. (33) found that positive formulations are better in this regard. In summary, studies have shown that descriptive norms are more successful when they target majorities rather than minorities. Furthermore, studies addressing *minorities* have shown that the effects were particularly negative when participants strongly identified with this minority group [e.g., (44); see also (42), for a similar negative result for the German subgroup]. Nevertheless, there are good reasons to continue research into descriptive norms among minorities. Firstly, there are only a handful of studies on this topic, which leaves us with an incomplete picture. Secondly, in many spheres of life, but especially in nutrition, only a minority of the population exhibits the desired behavior, such as consuming five servings of fruit and vegetables per day [(50); see (91), for further possible reasons]. However, it is precisely this group that needs to be reached. Hence, more research is needed. Note, however, that there are also studies that show no difference in effectiveness for either group [minority or majority, see, for example, (51) or (42)].

Another question that arises in connection with the success of descriptive norms is whether their success depends on (or interacts with) other factors such as *past behavior* [not to be confused with personal norms or dispositions; see (52)]. Research has shown that past behavior can either lead to *consistent* future behavior [the same or similar; see, for example, (53)] or to *inconsistent* future behavior. In the latter case, positive behavior in the past is offset by subsequent negative behavior [(54), for a meta-analysis; see also (55)]. Schultz et al. (55), for example, showed that feedback on the average energy consumption of neighbors reduced/increased personal future energy consumption. This depended on whether one's own energy consumption in the past was above/below this average [see also (33, 56, 57); and (58) on voting behavior]. Lalot et al. (59) also investigated the influence of past behavior, but with reference to majority/minority groups. The authors assessed the participants' previous environmentally friendly behavior (low, high) and examined its influence—using minority/majority descriptive norms—on their intention to behave in an environmentally friendly manner (i.e., to celebrate a green Christmas) in the future. Their results showed that for the majority group, past behavior was irrelevant (i.e., it always led to high scores in terms of the intention to behave in an environmentally friendly manner). For the minority group, however, past behavior only led to high scores in terms of intention if they had already behaved in an environmentally friendly manner in the past. Hence, in the context of a majority group, descriptive norms are sufficient to elicit environmentally friendly behavior, rendering past behavior irrelevant. Note, however, that most researchers assume that past behavior encompasses the concept of self-identification. However, in a follow-up study in which they separated self-identity from past behavior, Lalot et al. (60) found that the previously observed ineffectiveness of past behavior in the majority group may have been caused by interaction with self-identification. With low self-identification, past environmentally friendly behavior results in higher intentions than past environmentally harmful behavior. The opposite pattern can be observed with high self-identification. Studies have thus shown that past behavior is relevant to (future) intentions, even though its interaction with minorities/majorities descriptive norms has not yet been conclusively clarified.

In summary, it can be said that minority/majority descriptive norms (taking past behavior into account) is a promising intervention which could encourage people to buy healthier foods presented on social media (e.g., Instagram). But how can such descriptive norms be integrated into social media platforms such as Instagram? One possibility would be to implement them in a simple “Instagram feed post,” as Varni et al. (61) did. Apart from the fact that their study differs from ours in key aspects (no consideration of social norms, no manipulation of minority/majority, etc.), their implementation does not allow for consideration of past behavior. We therefore propose a different implementation, namely through the use of polls, that is, interactive features. In these polls, users are asked a closed question and presented with two predefined answers, for example, “Do you like Instagram? Yes/no.” After participating in a poll, the user immediately sees the results displayed in a horizontal bar. This bar, divided proportionally according to the distribution of real-time results, shows the respective percentages of yes/no answers. In addition, the percentages on the left (i.e., the yes answers) are usually displayed in green, while the percentages on the right (i.e., the no answers) are displayed in red (or pink). Last but not least, the side of the bar containing the user's selected answer is indicated by a subtle gray shading. This simple but informative feedback design not only allows the user to quickly and easily grasp both the opinion of the majority/minority and his/her own position on the issue, but also to assess the user's past behavior. To our knowledge, such polls—which implement social norms—have not yet been examined in terms of how they influence people's purchasing intentions with regard to food products presented on social media. For a better understanding of the structure of the manuscript, here a brief overview of the three studies conducted. Since Study 1—the baseline study—showed that the observed effects were primarily due to the visual characteristics of the poll, these were systematically manipulated in the subsequent studies. More specifically, in Study 2, the proportional representation of the results was removed, and in Study 3, the colored representation of the results was also removed.

## 2 Study 1

In our first study, we examine how participants' eating habits (past behavior) interact with minority/majority descriptive norms (manipulated via the poll) to influence purchase intentions for healthy/unhealthy food products advertised on a social media platform (like Instagram).

The visual features of the interactive poll are also kept as close to the original (as on Instagram) as possible. In line with the results discussed, we expected majorities to be most effective, but only if the descriptive norm is formulated positively and does not normalize undesirable behavior.

### 2.1 Method

#### 2.1.1 Participants

Two hundred twenty-seven students from the ZHAW Zurich University of Applied Sciences, who were recruited via its internal e-mail list and thus form an *ad-hoc* sample^7^, took part in this smartphone-based study. Their ages ranged from 19 to 52 years old (*M* = 25.6, *SD* = 5.3); whereby 60.4% of them were female. Participants could enter a drawing for an iPad (which 93.8% of them did); students of the School of Applied Psychology (9.7%) could instead receive course credit for their participation (which 40.9% of them did). All participants provided written informed consent^8^.

#### 2.1.2 Stimulus material

**Figure 1:**
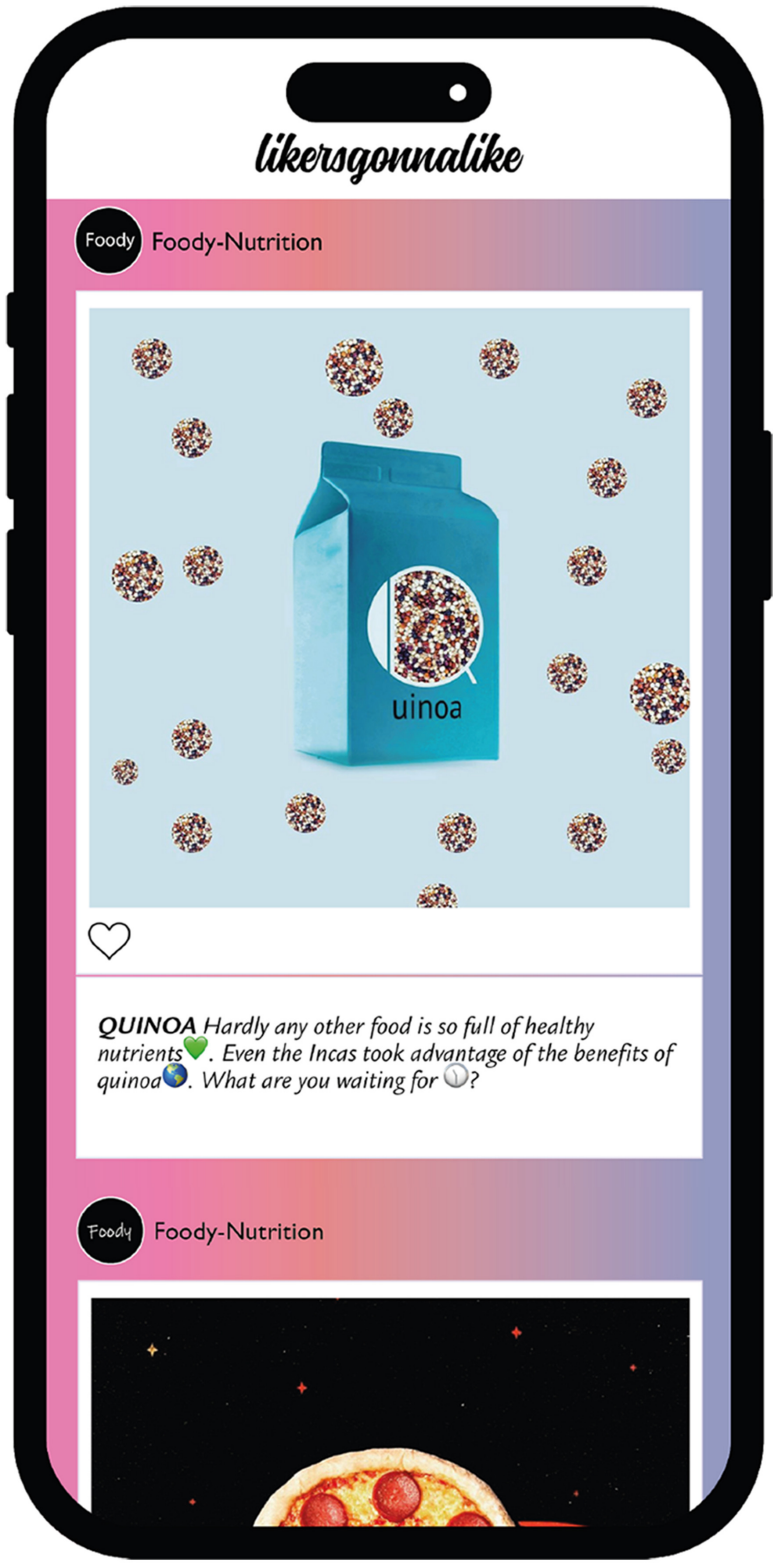
Example of the experimental interface on the artificial social media platform “likersgonnalike”.

Of the 10 foods that were presented to the participants and could be (hypothetically) purchased by them, 5 were *healthy* (Kale Chips, Go Nuts, Muh, Quinoa, Pro Drink) and 5 were *unhealthy* (Chok Chok, Snackerella, Planet Pizza, Ride, Farmer's Chips). This means that at the time of the study, healthy foods fell into Nutri-Score categories A and B, while unhealthy products fell into Nutri-Score categories D and E. Each food product was then embedded in a post (with the option to like it). The posts were then placed—per participant—in random order (to control for sequence effects) in a feed on an artificial social media platform called “likersgonnalike” (see Figure 1).

#### 2.1.3 Procedure and design

**Figure 2:**
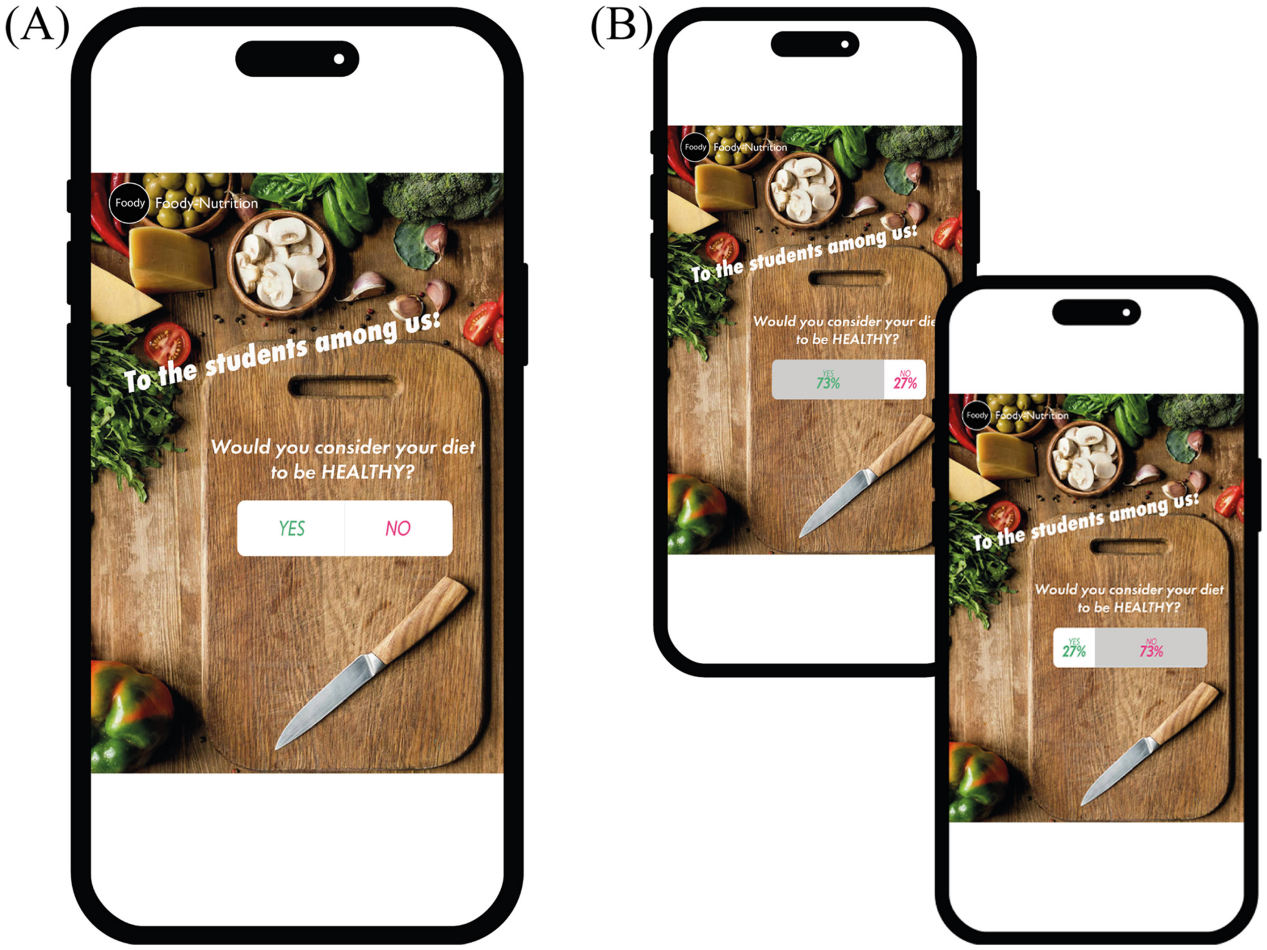
**(A)** Example of the poll question displayed on the “likersgonnalike” platform and **(B)** the respective answer from participants with the manipulated poll results. The images used have been modified from licensed Shutterstock images.

The smartphone-based study was implemented and conducted in Unipark (unipark.de). Through a cover story, participants were informed that the grocery store “Foody” was introducing its new food products on the social media platform “likersgonnalike.” However, before participants were presented with the products, they had to take part in a poll. In the poll they had to answer the question of whether their diet was healthy or unhealthy (random assignment; factor “framing”) on a binary answer scale (yes or no; see Figure 2A). Note that this manipulation (the framing of the question and the participant's response to it) enables us to record the participant's past behavior, while also taking into account positive/negative wording (i.e., “healthy” is positive wording, “unhealthy” is negative). In each of the two factor *framing* conditions, half of the participants were informed (again by random assignment) that their response belonged to the minority (27%), while the other half were informed that their response belonged to the majority (73%; factor “percentage”; see Figure 2B). Thereby the yes answers were displayed on the left-hand side of a bar and in green font color, while the no answers were displayed on the right-hand side of that bar and in red color. In addition, the percentages in the bar were displayed proportionally and the part of the bar representing the participants' answers was colored gray.

**Figure 3:**
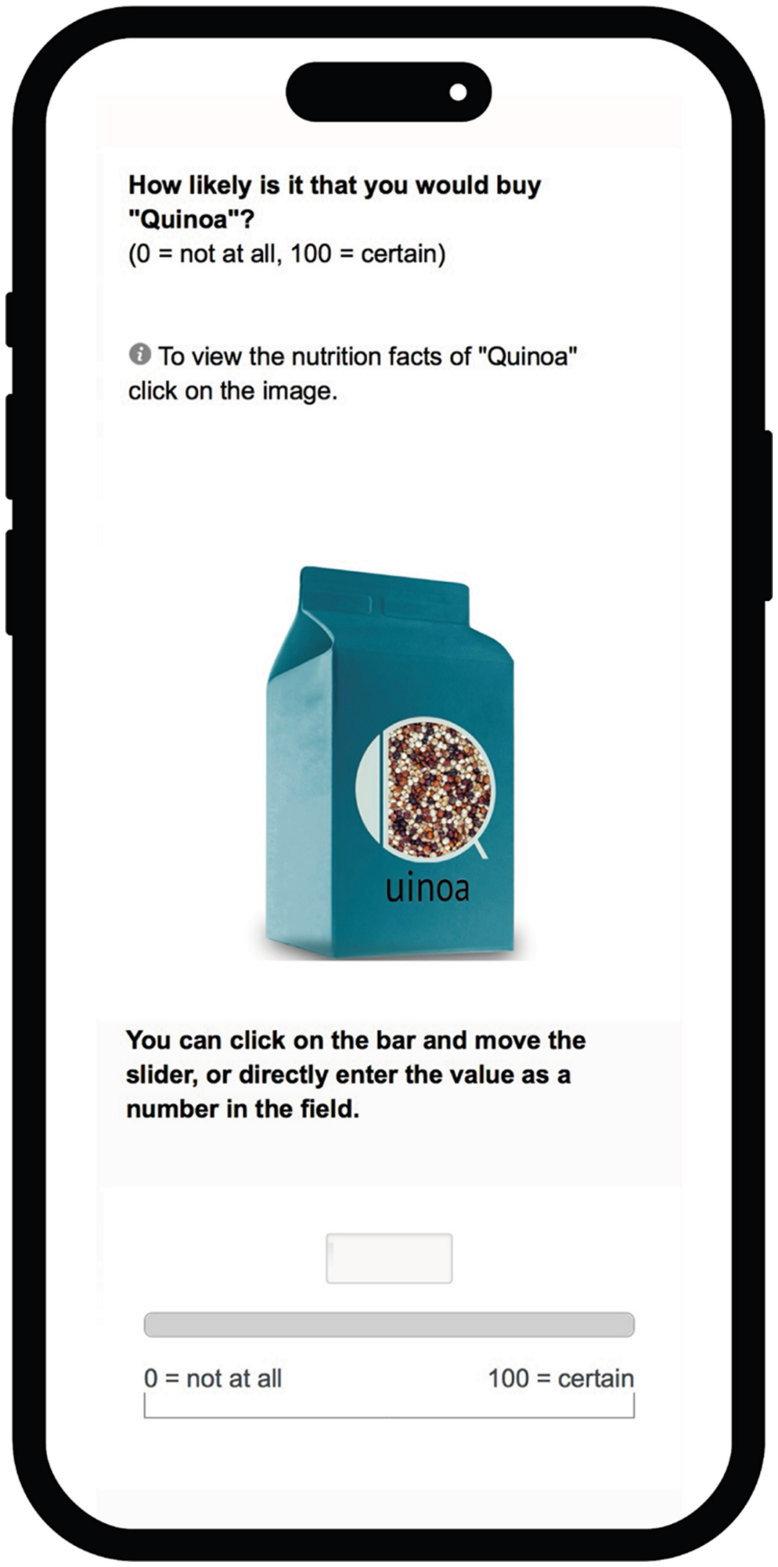
Illustrative example of the purchase intention rating used for the 10 products, shown here for Quinoa.

In addition to these four experimental conditions (2 framing conditions × 2 percentage conditions), we added a classic control group which did not receive the poll. After the participants in the four experimental conditions had completed the poll, all groups, including the control group that had not participated in the poll, were shown the feed with the 10 food products (5 healthy/5 unhealthy; factor “food product health”). Each of the products could be “liked.” After the participants had scrolled through the feed, the 10 food products were presented to them again. For each product, participants were asked to indicate the likelihood of purchase (purchase intention) on a scale from 0 to 100 (0 = not at all, 100 = definitely; see Figure 3^9^). Note that participants were able to view the nutritional information for each product (by clicking on the image). At the end of this study, participants' social media behaviors (e.g., which platforms they used and how often, e.g., daily, weekly, etc.) and demographic data (e.g., age, income, etc.) were recorded.

### 2.2 Results and discussion

Participants with reaction times (RTs) faster than 180 ms or slower than 1,800 ms were excluded from the data analysis (4.6% in total). Data in all studies were analyzed using IBM SPSS (version 29) Statistics.

#### 2.2.1 Purchase intention

We hypothesized that a *person's eating habits* (past behavior) might play a decisive role in the effect of descriptive norms and the reference to minority or majority groups. Therefore, the framing question and participants' answers to it were combined to create two groups with a diet that is either healthy or unhealthy (factor “diet”). Hence, we calculated 2 (product health [within-subject]: healthy vs. unhealthy) × 2 (left-side percentage [between-subject]: 27% vs. 73%) mixed ANOVAs on purchase intention (in %), separately for the two diet groups^10^.

##### 2.2.1.1 Healthy diet group

**Figure 4:**
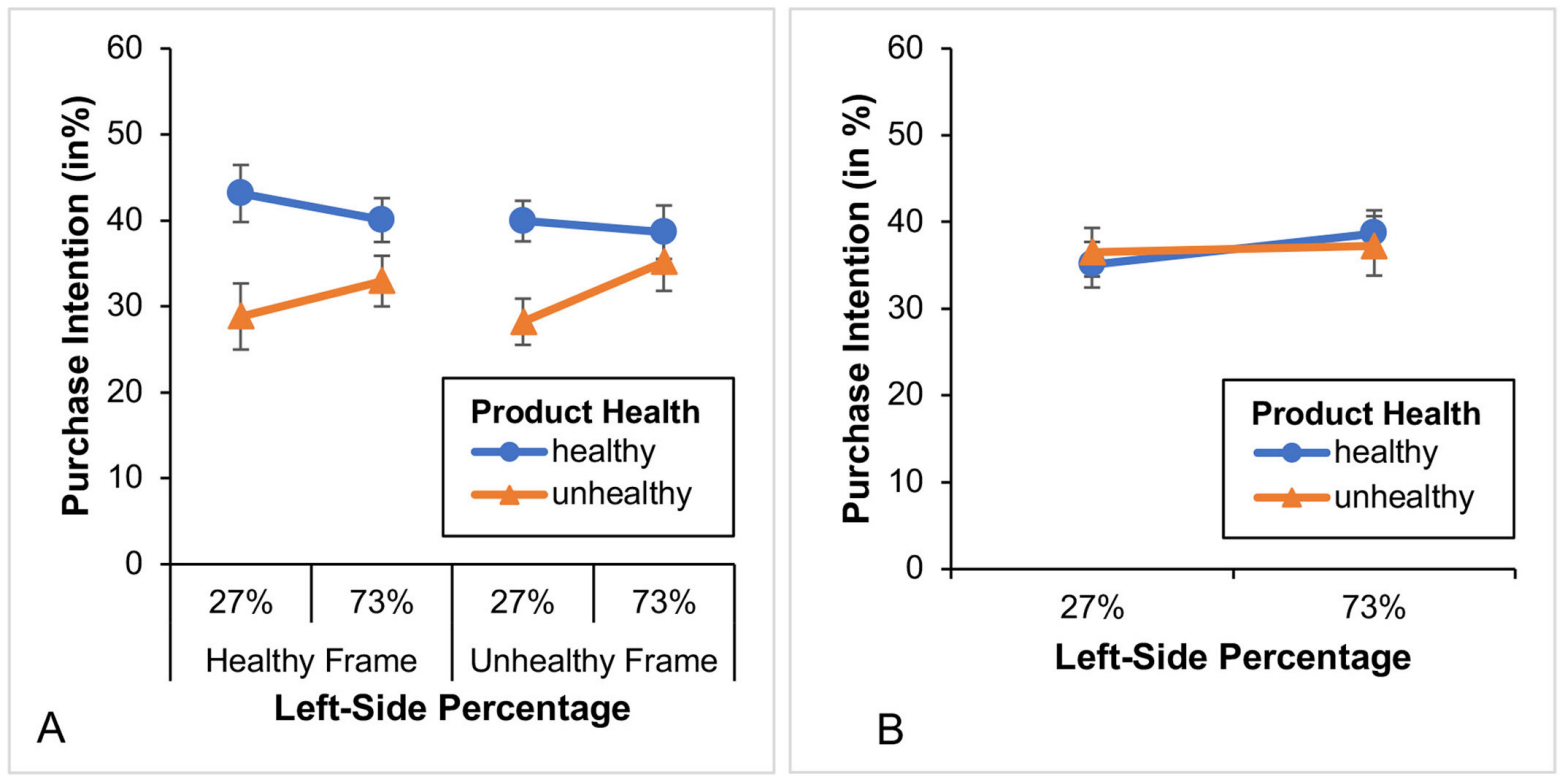
**(A)** Illustrates the purchase intention and the two different framing conditions for the healthy diet group while **(B)** illustrates the purchase intention for the unhealthy diet group without a subdivision according to the framing conditions. Only left-side percentages are presented, as the corresponding right-side values are complementary. Error bars show standard errors.

In this group (*n* = 133), we found a significant main effect of food product health, *F*_(1, 131)_ = 27.04, *p* < 0.001, η*p*^2^ = 0.171, but not of percentage, *F*_(1, 131)_ = 0.51, *p* = 0.476. Furthermore, we also found a significant Product health × Left-side percentage interaction, *F*_(1, 131)_ = 4.66, *p* < 0.05, η*p*^2^ = 0.034 (see Figure 4A). Although participants bought overall fewer unhealthy products than healthy ones, this effect was more pronounced when the 27% appeared on the left (i.e., was placed on the left side). To further explore this interaction, we conducted 4 *t*-tests (2 paired and 2 unpaired). We found (Bonferroni-corrected results) that only the left-side 27%, but not the left-side 73%, showed a significant difference between the intention to purchase healthy and unhealthy products [left-side 27%: *t*_(66)_ = 4.78, *p* < 0.001; left-side 73%: *t*_(65)_ = 2.391 *p* = 0.08; explaining the interaction]. Regarding the intention to purchase unhealthy products *t*_(131)_ = −1.74, *p* = 0.336 (orange line) or healthy products *t*_(131)_ = 0.73, *p* = 1.00 (blue line), we also found no significant difference between the left-side 27% and left-side 73%.

##### 2.2.1.2 Unhealthy diet group

In this group (*n* = 49), both the main effects (product health and left-side percentage) and their interaction were not significant (all *F-values* ≤ 0.316 and all *p-values* ≥ 0.576; see Figure 4B).

#### 2.2.2 Liking

We wanted to know whether (in addition to our manipulations) the *liking* of a food product had an influence on purchase intention. Of the total of 2,270 products presented (10 products per participant), 460 products were “liked,” which corresponds to an average of 20.3%. The proportion of healthy products that were liked was 51.5%, which was almost as high as the proportion of unhealthy products that received a like (48.5%).

To examine the association between product liking (yes/no) and purchase intention (yes/no^11^), six chi-square tests of independence were conducted. That is, one test per group (unhealthy diet/healthy diet/control) × food product purchase intention (all products/only healthy products/only unhealthy products) combination. The analysis revealed significant relationships regarding all combinations (all χ^2^-values ≥ 13.53, all *p-values* < 0.001). For example, for the combination “Healthy diet × All products” (*n* = 1,330; note that *n* stands for the number of products), we observed a chi-square value of: χ^2^(1) = 201.84, *p* < 0.001, ϕ = 0.39. This means that participants who liked the product (healthy or unhealthy) were more likely to buy it or vice versa.

#### 2.2.3 Social media use

**Figure 5:**
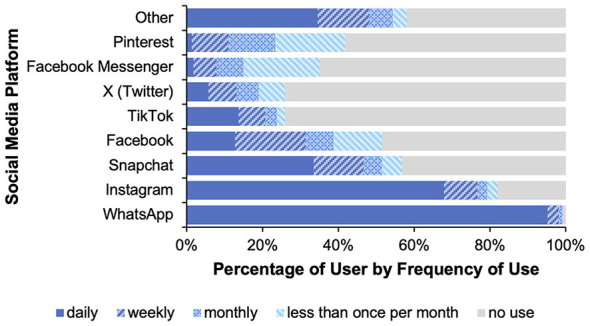
It shows the percentage-based usage of various social media platforms by frequency (daily, weekly, monthly, less than once per month, and no use). The most frequently mentioned “other” platforms were YouTube, Telegram, and Reddit.

The analysis of participants' social media use showed that WhatsApp and Instagram were most frequently used on a daily basis (see Figure 5).

#### 2.2.4 Discussion

We found a difference in purchase intention, but only in the *healthy diet* group. In addition, the effect was more pronounced when the percentage of 27% was presented on the left side. Hence, it was irrelevant what significance the percentage of 27% (or 73%) had, that is, whether one actually belonged to the minority group or not (as indicated by participants' answers). These results cannot be attributed to either a norm-consistent influence or a contrasting effect that could be explained, for example, by reactance. We therefore hypothesize that the graphical features of the poll results influenced purchase intention (this hypothesis will be tested in Study 2 and 3). These graphical features are, more specifically, the proportional representation (27% = short bar/73% long bar) and/or the color coding of the answers (yes = green/no = red). The lack of effect in the group following an unhealthy diet can possibly be attributed to their previous behavior, as this increases the likelihood of consistent behavior in the future (59). It therefore seems plausible that people who have not considered healthy eating to be important in the past are less likely to respond to our intervention. This is consistent with the idea that past behavior not only reflects previous decisions but is also relevant for future decisions (59). Last, but not least, there was a significant positive relationship between the liking of a product and its purchase intention. However, unfortunately this was true for the unhealthy as well as the healthy products. Considering these unexpected results, the *visual features of the poll* will be further researched and systematically manipulated.

## 3 Study 2

The first manipulation, given the visual features of the poll, is the removal of the “proportional representation” of the poll results. Proportional representation means that the percentage is reflected in the length of the bar (e.g., high percentage = longer bar). This allows poll results, for example, to be interpreted more clearly and accurately (62). Swiss voters, for example, are familiar with this type of presentation. The booklet summarizing the information on the popular initiative uses lines of varying lengths to show how many people in the two chambers (National Council and Council of States) of the Swiss Parliament say yes or no to the initiative. Accordingly, when the proportional representation is removed, people can no longer rely on perceptual heuristics such as the length of the bars to extract quantitative meanings. This can lead to different judgments. Therefore, the removal of the proportional representation is intended to test whether the unexpected results in Study 1 were influenced by this.

### 3.1 Method

#### 3.1.1 Participants

Two hundred twenty-three students from the ZHAW Zurich University of Applied Sciences took part in this smartphone-based study. Their ages ranged from 19 to 52 years old (*M* = 25.9; *SD* = 5.5), whereas 62.6% were female. Participants could enter a drawing for an iPad (which 85.7% did); students of the School of Applied Psychology (12.1%) could instead receive course credit for their participation (which 81.8% did). All participants provided written informed consent.

#### 3.1.2 Stimulus material, procedure, and design

**Figure 6:**
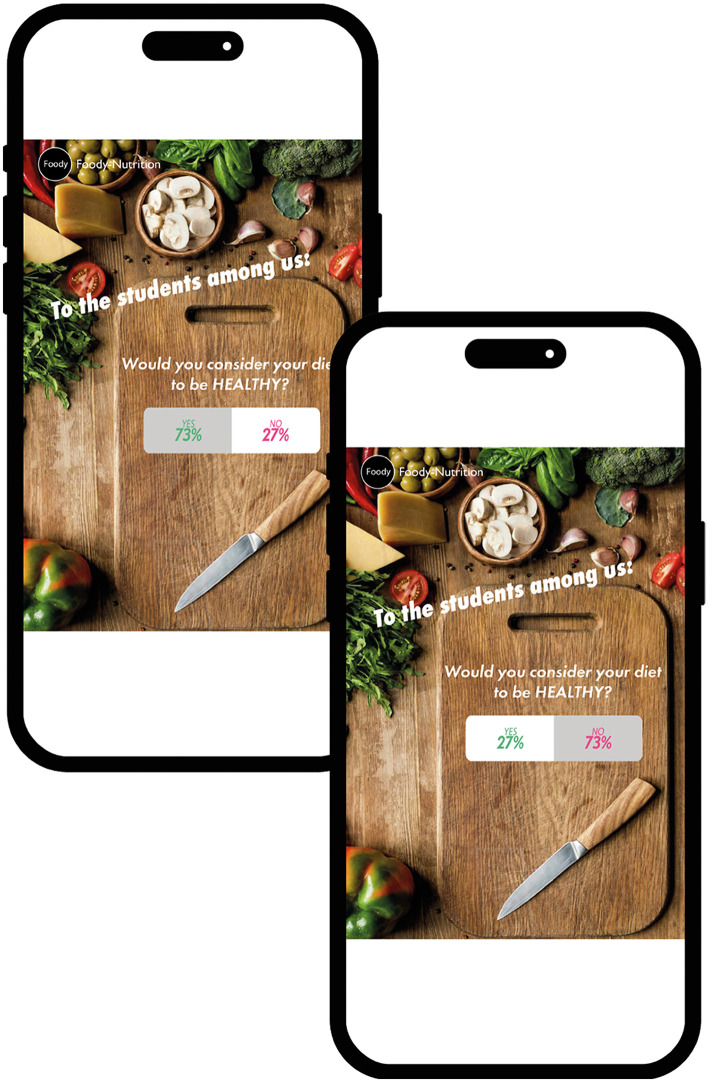
Poll results as presented in Study 1, with the proportional representation removed. The images used have been modified from licensed Shutterstock images.

We used the same stimulus material, procedure, etc. as in Study 1^12^, with the following exception: the percentages were now represented by two bars of equal length (see Figure 6).

### 3.2 Results and discussion

Reaction times (RTs) faster than 180 ms or slower than 1,800 ms were excluded from the data analysis (2.8% in total).

#### 3.2.1 Purchase intention

We again calculated two 2-factorial mixed ANOVAs, separately for the two diet groups. While the independent within-factor “product health” (healthy vs. unhealthy) remained the same as in Study 1, the other independent between-subject factor, “percentage selected,” now symbolizes something different than in Study 1 (see explanation below).

##### 3.2.1.1 Healthy diet group

**Figure 7:**
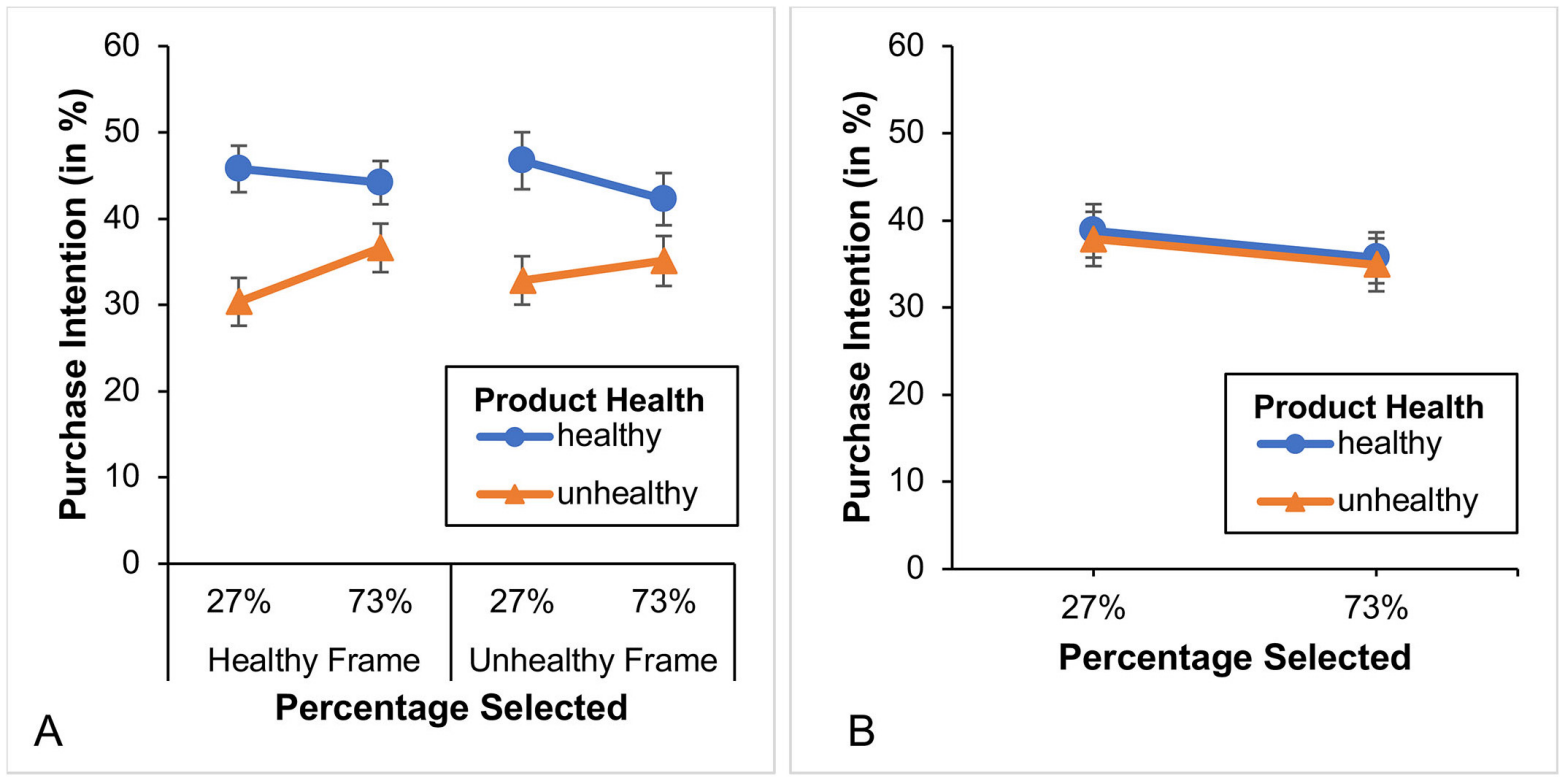
**(A)** Illustrates the purchase intention for the healthy diet group and the two different framing conditions while **(B)** illustrates the purchase intention for the unhealthy diet group without a subdivision according to the framing conditions. Only selected percentages are presented, as the corresponding values are complementary. Error bars show standard errors.

A word about the factor “percentage selected.” In Study 1, we calculated the average of the different framings based on whether or not the percentage of 27% was displayed on the left (i.e., it did not matter whether participants had selected it or not). We did this because the underlying interaction pattern was the same (see Figure 4A). However, upon visual inspection of the data from Study 2 (see Figure 7A), we found that the underlying pattern was defined differently this time. That is, it was not the position of the percentage of 27% which mattered, but rather the fact of whether or not the percentage of 27% was selected. Therefore, in Study 2, we averaged differently across the different framings, resulting in a factor called “percentage selected.” By analyzing the data in this way^13^ we again found in this group (*n* = 152) a significant main effect of product health, *F*_(1, 150)_ = 42.54, *p* < 0.001, η*p*^2^ = 0.221, while again the main effect of percentage selected was not significant *F*_(1, 150)_ = 0.10, *p* = 0.756. Furthermore, we again found—though this time with a different underlying meaning—a significant Product health x Percentage selected interaction, *F*_(1, 150)_ = 4.67, *p* = 0.032, η*p*^2^ = 0.030 (see Figure 7). That is, participants with healthy diets showed a higher purchase intention for healthy compared to unhealthy foods, and this effect was more pronounced when the percentage of 27% was selected (i.e., marked with a gray bar). Again, to further investigate this interaction, we conducted 4 *t*-tests (2 paired and 2 unpaired). We found (Bonferroni-corrected results) that both selected percentages (27% and 73%) showed a significant difference between the purchase intention for healthy and unhealthy products [selected 27%: *t*_(73)_ = 6.26, *p* < 0.001; selected 73%: *t*_(77)_ = 3.04, *p* < 0.05], with the former effect being stronger (which explains the interaction). However, again there was no significant difference in purchase intention for unhealthy products (orange line) and healthy products (blue line; all *t*-*values* < 1.552 and all *p*-*values* ≥ 0.490) with regard to the selected percentages (27% vs. 73%).

##### 3.2.1.2 Unhealthy diet group

For this group (*n* = 63), again neither the main effects (product health and percentage selected) and their interaction were significant (all *F*-*values* ≤ 0.679 and all *p*-*values* ≥ 0.413).

#### 3.2.2 Liking

As in Study 1 we wanted to know whether (in addition to our manipulation) the liking of a product had an influence on purchase intention. Of the 2,730 products in total (10 products/participant), 564 products were “liked,” which corresponds to an average of 20.7%. The proportion of healthy products that were liked was 54.1%, which was slightly higher than the proportion of unhealthy products that received a like (45.9%).

To examine the association between product liking (yes/no) and purchase intention (yes/no) (see text footnote 11), six chi-square tests of independence were conducted. That is, one test per group (unhealthy diet/healthy diet/control) × food product purchase intention (all products/only healthy products/only unhealthy products) combination. The analysis revealed a significant relationship regarding all combinations (all χ^2^-values ≥ 19.62, all *p*-*values* < 0.001). For example, for the combination “Healthy diet × All products” (*n* = 1,520; note that n stands for the number of products) the chi-square was: χ^2^(1) = 184.12, *p* < 0.001, ϕ = 0.35. This means that participants who liked the product (healthy or unhealthy) were more likely to buy it or vice versa.

#### 3.2.3 Social media use

**Figure 8:**
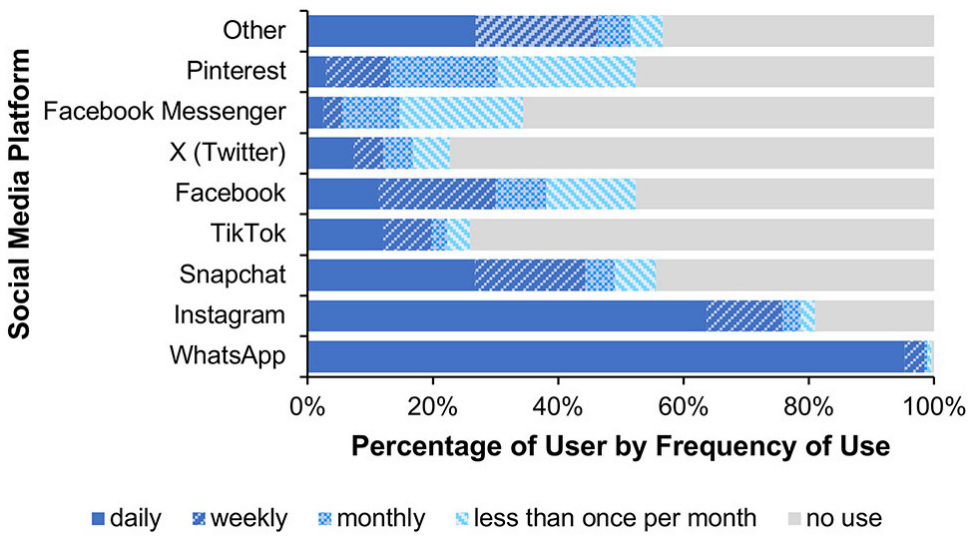
This figure shows the percentage-based usage of various social media platforms by frequency (daily, weekly, monthly, less than once per month, and no use). The most frequently mentioned “other” platforms were Reddit, YouTube, and Telegram.

The analysis of participants' social media use showed that in Study 2, WhatsApp and Instagram were most frequently used on a daily basis, as was the case in Study 1 (see Figure 8).

#### 3.2.4 Discussion

As in Study 1, we found that in the healthy diet group, purchase intention for healthy products was higher than for unhealthy products, and that this effect was more pronounced at the selected percentage of 27%. However, due to the removal of the *proportional representation* of the poll results, it can be seen that the effect has shifted from the “27%” shown on the left to the “27%” that was actually selected (shown with a gray bar). Consistent with previous findings, no significant effects were found for the unhealthy diet group. Interestingly, the colors of the percentages did not seem to have any influence, as the selected 27% showed the same effect regardless of whether they were colored green or red. Nevertheless, we eliminated this visual feature in Study 3 to ensure that the observed effect is not dependent on color.

## 4 Study 3

Study 3 further investigates the potential influence of visual features of polls by the additional removal of the color components. While the effect observed in Study 2 for the selected 27% appeared to be independent of color, the observed shift in pattern suggested that this visual feature could nevertheless have a significant influence. In particular, red and green are known to be carriers of *affective* meanings. Green appears to be associated with positive meanings and elicits approach responses, while red appears to be associated with negative meanings and tends to elicit avoidance responses (63, 64).

### 4.1 Method

#### 4.1.1 Participants

One hundred forty-nine students from the ZHAW Zurich University of Applied Sciences took part in this smartphone-based study. Their ages ranged from 20 to 51 years old (*M* = 26; *SD* = 5.4), and 52.3% were female. All participants gave informed consent. As an incentive, participants could take part in a drawing for an iPad (which 85.2% did), and students of the School of Applied Psychology (9.4%) could receive course credit instead (which 57.1% overall did).

#### 4.1.2 Stimulus material, procedure, and design

**Figure 9:**
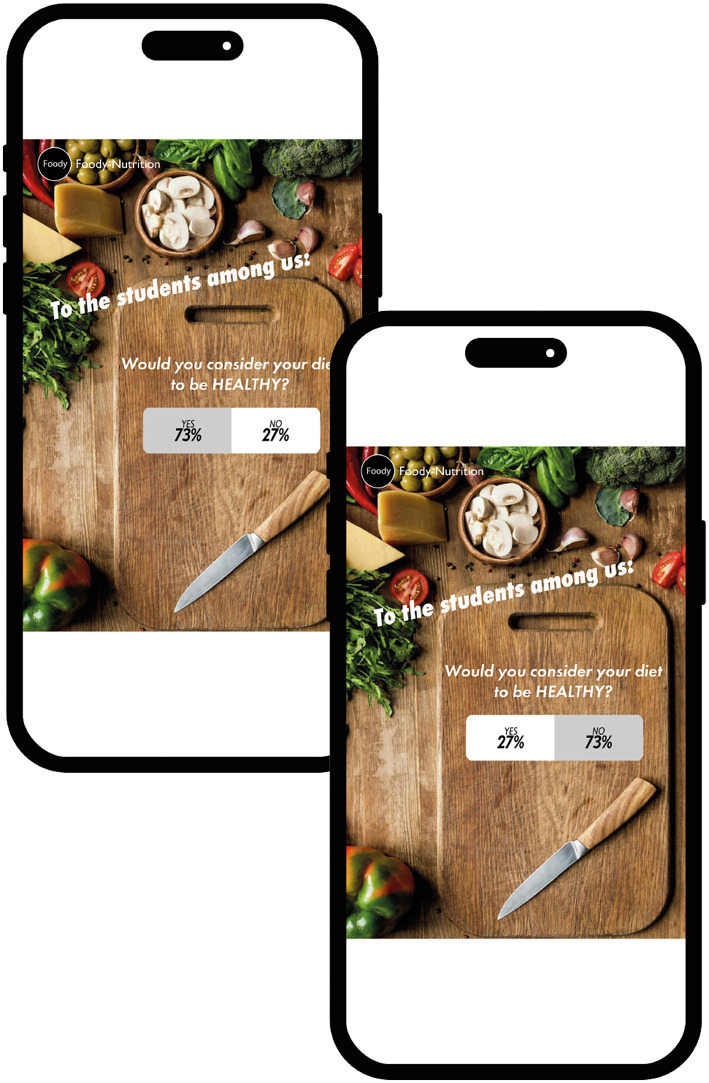
Poll results as presented in Study 1, with the proportional representation and colors removed. The images used have been modified from licensed Shutterstock images.

We used the same stimulus material, procedure, etc. as in Study 2 (again with a new study sample; see also footnote 10) with the following exception: we removed the color representation of the choices *yes* (green) and *no* (red; see Figure 9).

### 4.2 Results

Reaction times (RTs) faster than 180 ms or slower than 1,800 ms were excluded from the data analysis (5.7% in total).

#### 4.2.1 Purchase intention

Visual inspection of the data showed that no interactions were to be expected, so the way in which the percentages (“left-side percentage” or “percentage selected”) are combined should not matter. We communicate the results of the analysis in the text using the “left-side percentage” factor as in Study 1.

##### 4.2.1.1 Healthy diet group

**Figure 10:**
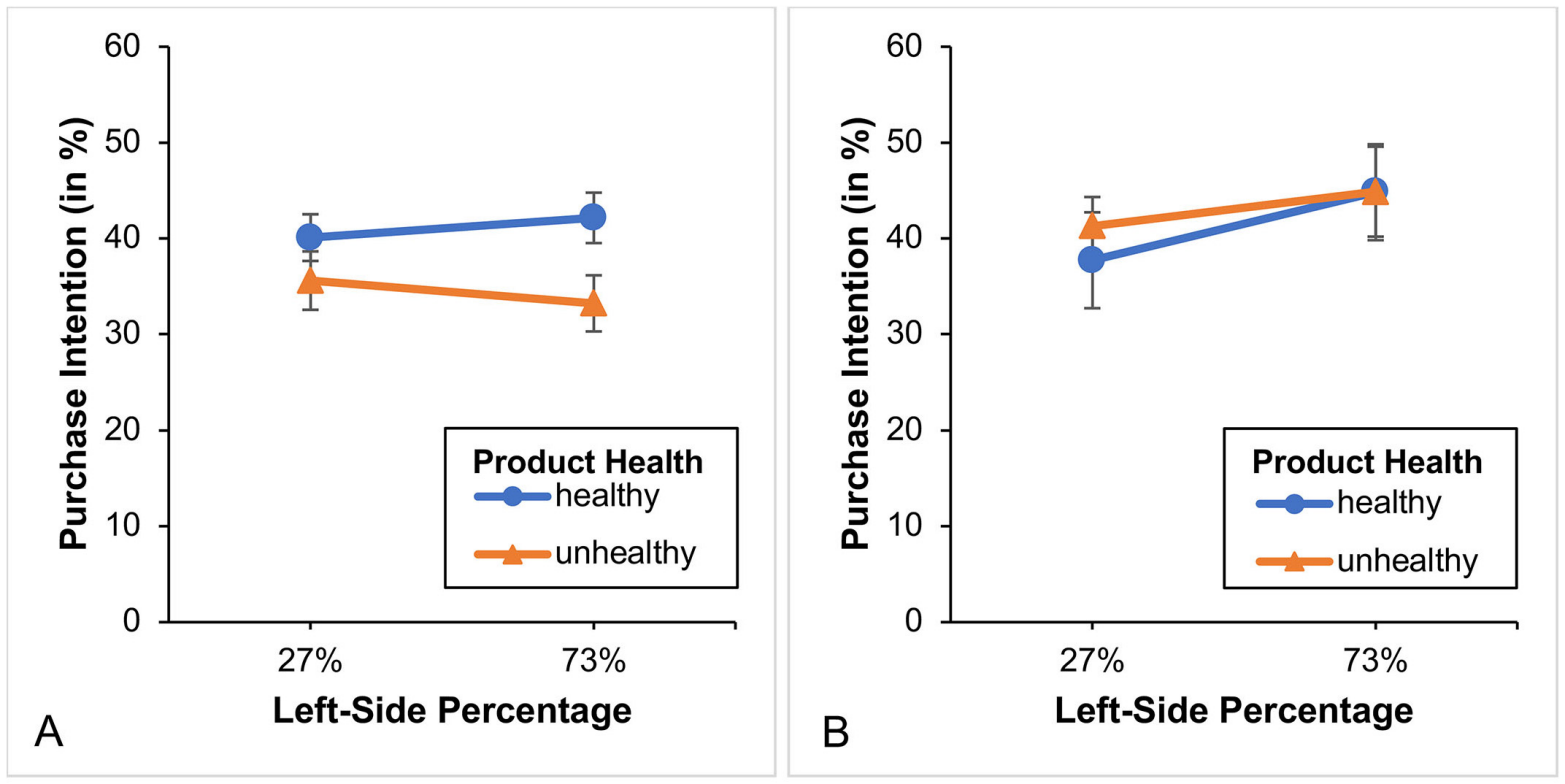
**(A)** Illustrates the purchase intention for the healthy diet group while **(B)** illustrates the purchase intention for the unhealthy diet group. Only left-sided percentages are presented, as the corresponding values are complementary. Error bars show standard errors.

In this group (*n* = 82) we again found a significant main effect for product health, *F*_(1, 80)_ = 7.46, *p* < 0.01, η*p*^2^ = 0.085. Note that this effect (see partial eta) is much smaller than that in Study 1 or 2 (i.e., the difference in purchase intention between healthy and unhealthy foods is smaller). Furthermore, we found neither a significant main effect for left-side percentage, *F*_(1, 80)_ = 0.00, *p* = 0.963, nor a significant Product health × Left-side percentage interaction, *F*_(1, 80)_ = 0.82, *p* = 0.369 (see Figure 10). For the first time, the poll results or its visualization no longer have any impact.

##### 4.2.1.2 Unhealthy diet group

In line with Study 1 and Study 2, for this group (*n* = 34) again both main effects (product health and left-side percentage) and their interaction were not significant (all *F*-*values* ≤ 0.969 and all *p*-*values* ≥ 0.332).

#### 4.2.2 Liking

As in Study 1 and 2 we wanted to know whether (in addition to our manipulation) the liking of a product has an influence on the purchase intention. Of the 1,490 products in total (10 products per participant), 263 products were “liked,” which corresponds to an average of 17.7%. The proportion of healthy products that were liked was 48.7%, which was slightly lower than the proportion of unhealthy products that received a like (51.3%).

To examine the association between product liking (yes/no) and purchase intention (yes/no) (see text footnote 11), six chi-square tests of independence were conducted. That is, one test per group (unhealthy diet/healthy diet/control) × food product purchase intention (all products/only healthy products/only unhealthy products) combination. The analysis revealed a significant relationship regarding all combinations (all χ^2^-values ≥ 10.72, all *p*-*values* < 0.001). For example, for the combination “Healthy diet × All products” (*n* = 820; note that *n* stands for the number of products) the chi-square was: χ^2^(1) = 130.68, *p* < 0.001, ϕ = 0.40. This means that participants who liked the product (healthy or unhealthy) were more likely to buy it or vice versa.

#### 4.2.3 Social media use

**Figure 11:**
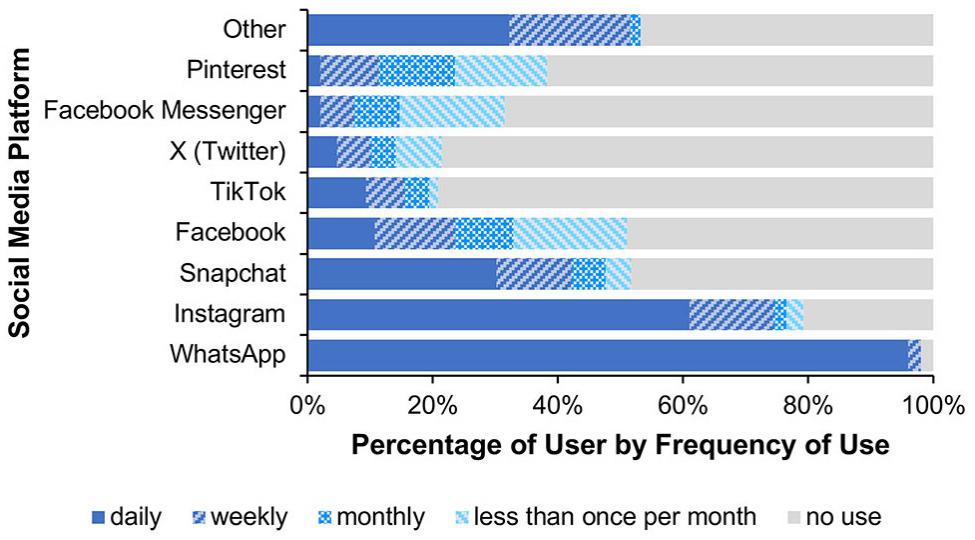
It shows the percentage-based usage of various social media platforms by frequency (daily, weekly, monthly, less than once per month, and no use). The most frequently mentioned “other” platforms were YouTube, Signal, and LinkedIn.

The analysis of participants' social media use showed that in Study 3, WhatsApp and Instagram were most frequently used on a daily basis, as in Studies 1 and 2 (see Figure 11).

#### 4.2.4 Comparison of control groups

**Figure 12:**
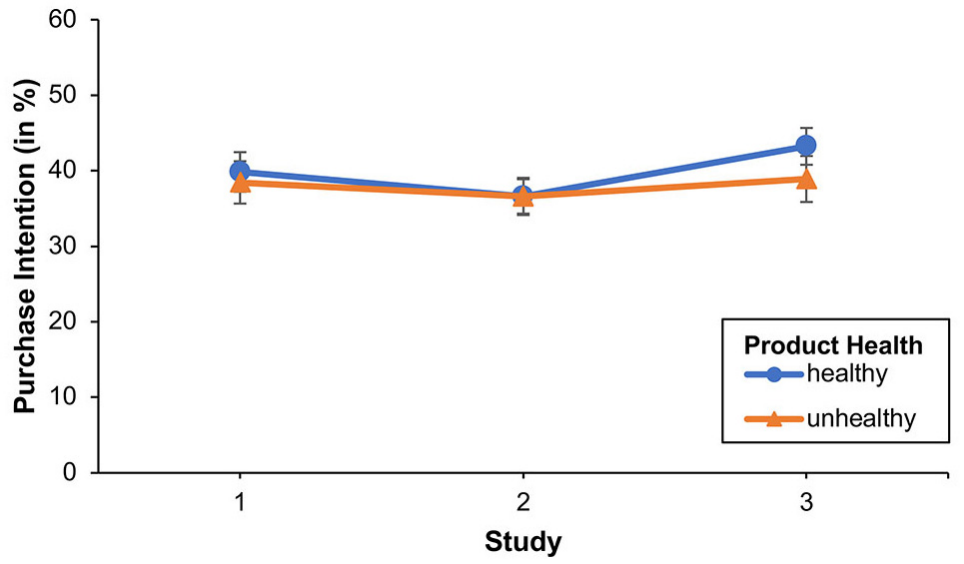
The figure illustrates the purchase intention for healthy and unhealthy food products across the control groups of the three studies. Error bars show standard errors.

One could argue that the results of the three studies are due to the different study populations. To rule out this possible explanation, we compared the control groups of the three studies with each other. To this end, we conducted a 2 (product health [within-subject]: healthy vs. unhealthy) × 3 (study [between-subject]: study 1 vs. study 2 vs. study 3) mixed ANOVA on purchase intention. We thereby found neither significant main effects of product health or study, nor a significant Product health × Study interaction effect, all *F-values* ≤ 1.133, and all *p-values* ≥ 0.325 (see Figure 12). The results show that the control groups in all three studies are comparable.

#### 4.2.5 Discussion

As in Studies 1 and 2, we again found a significant main effect for product health in the healthy diet group, although this effect was less pronounced than in Studies 1 and 2. Furthermore, due to the removal—in addition to the *proportional representation*—of the *color representation* of the poll results, we were unable to observe a significant interaction for the first time. The results for the unhealthy diet group, as well as, for example, the correlation between liking and purchase intention, correspond to those found in Study 1 and 2. Importantly, the comparability of the control groups shows that the effect of product health observed in all three studies in the healthy diet group was indeed caused by the dietary habits of the participants (healthy vs. unhealthy diet). Therefore, past behavior seems to matter.

## 5 Conclusion and policy implications

Overall, in all three studies, we found a significant difference (of about 7%) in the intention to purchase healthy and unhealthy products in conditions where participants followed a *healthy diet*. This effect was to be expected, as *past behavior* can increase the likelihood of consistent behavior in the future (59). However, in Studies 1 and 2 there were conditions under which this effect could be doubled (about 14%). While in Study 1 these were the conditions in which the “27%” was placed on the left, in Study 2—in which the proportional representation was removed—these were the conditions in which the “27%” was selected by the participants. In Study 3, where the colors as well as the proportional representation were removed, no such doubling of the gap in purchase intention between healthy and unhealthy products could be observed under any conditions. Therefore, a healthy diet and proportional and/or color coding appear to be relevant to the occurrence of the observed effects.

The question is, can our observed effects be explained by the research findings presented in the introduction or the assumptions derived from them? In this regard, it has been shown, for example, that *majorities* are the most effective reference group (25, 38, 47). Thereby it is irrelevant whether the behavior is desirable (e.g., vegetable consumption) or undesirable [e.g., smoking; (33, 55)]. However, regarding our observed effects, we were unable to identify any majority effect (e.g., under a healthy framing, for example, a 73% “yes” result should have led to a reduction in purchase intention for unhealthy products and/or an increase in the purchase intention for healthy products). Our observed effects cannot be explained by minority norms either, that is, that the influence of minorities tends to be negative, especially in cases of strong identification [as found by Richter et al. (42), and by Stok et al. (44)]. Therefore, social norms alone cannot explain the effects we observed. But what if they are combined with *past behavior*, as in Lalot et al. [(59); see introduction]? On the one hand, Lalot et al. postulated that in the case of a majority norm, past behavior should be irrelevant and that future behavior should always be guided by the majority. On the other hand, they postulated that in the case of a minority norm, past behavior should lead to consistent future behavior. When we combine their predictions with our observed effects, we see that they cannot be confirmed regarding the majority norm (no effect among participants who followed an unhealthy diet), while they were confirmed regarding the minority norm. Therefore, even the combination of social norms and past behavior can only partially explain our results. However, the observed effects can be explained well by our experimental manipulations (visual features), as the purchase intention for healthy and unhealthy products did not differ significantly in the control groups of all three studies. Hence, we rule out an explanation of the observed effects by existing product preferences. It is noteworthy that the manipulated health majority corresponds to the participants' actual past dietary behavior. In all three studies, approximately 73% reported maintaining a healthy diet (Study 1: 73%; Study 2: 71%; Study 3: 71%). This correspondence enhances the relevance of our observed effects, as it suggests that the manipulations not only had an effect but also reflected the participants' actual descriptive norm. In summary, to explain our effects observed, we exclude explanations based on *descriptive norm feedback*.

Instead, we focus on the poll's *visual features*. In *product marketing*, the importance of visual product features (e.g., its color) compared to other relevant, factual product features (e.g., its quality) and their primary consideration in a decision has long been recognized (*visual dominance hypothesis*). For example: all of us have at some point purchased a bottle of wine based on the appearance of the label (visual feature), even though there may have been better alternatives (e.g., better grapes or combinations thereof; factual feature). Accordingly, Jansson-Boyd and Bright (65) have stated: “These days the visually based design features are engineered to entice consumers given the strong belief that purchase decisions can be manipulated by aesthetic components of the visual input” (p. 51). The fact that visual features also play a decisive role outside of product marketing, for example in dietary decisions, was demonstrated in the study by Schulte-Mecklenbeck et al. [(66); see also (67–69)]. In the study by Schulte-Mecklenbeck et al. (66), participants had to make several decisions between two lunch dishes, with visual information (pictures of the dishes) and factual information (name, price, and nutritional information) presented as decision aids. The results showed that participants made their decisions *primarily* based on visual information. Although research suggests that the systematic manipulation of colors, graphics, logos, and layout influences consumers' purchasing intentions/decisions, it is still unclear exactly how visual features do this (70). Regarding communicating quantitative information, it is generally recommended to maintain graphic integrity through a visual representation of the results (62). Similarly, Gigerenzer et al. (71) emphasize that numerical representation of data could be too difficult, whereas visual representations could facilitate processing. Through our studies, we have attempted to clarify the role of visual features in connection with descriptive norm feedback.

We found that the systematic removal of visual features (such as *proportionality* in Study 2 and *colors* in Study 3) led to a change in the effects observed in Study 1^14^ (Study 2), or even to their complete absence (Study 3). Note that there was no additive effect of the two design features, as the effects of Study 1 and 2 are comparable. Regarding the violation of graphic integrity by simply removing *proportionality*, it is interesting to note that this did not eliminate the effect observed in Study 1 but rather changed it. It was no longer evident in relation to the “left 27%,” but rather in relation to the “selected 27%.” It appears that if the bar length is no longer available as representative information due to the removal of proportionality, it can no longer be used to obtain quantitative information from the poll results. As a result, the salience of the norm information decreases, as the discrepancy between the two norms is no longer apparent from the visual information, but only from the interpretation of the percentage values. This may have shifted the focus in Study 2 to the “selected 27%.” However, this would mean that the semantic distinction between the formulations (healthy vs. unhealthy) was no longer considered when interpreting the poll results and that only the percentages of the minority were considered relevant. On the other hand, the observed main effect of product health among participants who follow a healthy diet suggests that the meaning of the wording was considered and that our operationalization of the categories “healthy” and “unhealthy” remains valid. To reconcile the effects observed in Studies 1 and 2 and their absence in Study 3 a *fundamental theoretical framework* is needed. That is, a framework that—as in our case—combines “social norm feedback” and “visual features.” One of the best-known frameworks into which our studies or their results could be integrated is the EAST framework developed by the Behavioural Insights Team (BIT) (72). It classifies possible interventions, or in their case so-called nudges to change human behavior, into: “Simple,” “Attractive,”^15^ “Social,” and “Timely.” Our visual features would thereby fall into “Attractive,” and social norms into “Social.” However, to our knowledge, there are only a few studies that examine the effect of a combination of interventions from different areas [see also (73)], and their results paint a mixed picture. Some clearly show an advantage of combining interventions or nudges [see, for example, (74, 89)]. However, other studies [such as (75)] show that a combination of (digital nudging) interventions, such as a default option and a social norm, can even backfire. Our studies and results can probably be classified in the latter category.

Despite the interesting findings of our three studies, there are potential limitations. Firstly, the use of a *student sample*. The advantage of its easy accessibility is offset by the disadvantage of all *ad hoc* samples—due to the lack of random selection—namely the lack of generalizability of the results. However, most of the ZHAW students have already completed initial training before beginning their studies. Therefore, according to the “proximal similarity” model (76), our results can be generalized to, for example, people of similar age, with similar digital skills, in similar financial situations, and in similar occupations (e.g., computer scientists, carpenters, etc.). However, it is not possible to generalize the results, for example, to older people or those who are less digitally savvy (similar considerations apply with regard to environments and times). However, in order to confirm these assumptions, replications with proposed groups of people are essential. Secondly, although *effects* were observed in participants following a healthy diet, this was unfortunately not the case for participants following an unhealthy diet. This could have been caused by the way we operationalized “past behavior.” In this context, we must critically examine whether our single self-perceived diet question (with its binary response format) actually measured what we wanted to measure (i.e., past behavior) and/or whether we should have used a validated instrument with multiple questions instead of measuring past behavior with a single question. Our operationalization was chosen to mimic the poll format used on social media platforms such as Instagram, thereby also ensuring user familiarity. In our defense, Loftfield et al. (77), for example, have shown that subjective and objective markers (e.g., fruit and vegetable consumption, fast food, blood pressure, etc.) can be adequately measured by a single indicator or question about overall dietary quality [“In general, how healthy is your overall diet?”; see also (78–81) regarding single-item measures]. However, future studies must show whether our operationalization's were correct and whether the same effects can be produced with other operationalization's. Finally, it might have been useful to record participants' subjective perceptions of the healthiness of the products. This could clarify whether the objective healthiness of the products and the participants' subjective perceptions of the healthiness of the products coincide or not. This would allow to investigate whether the lack of an effect of descriptive norm feedback is simply due to an incorrect categorization of the products as healthy or unhealthy. However, a stronger focus on the participants' subjective perception of the healthiness of the products carries the risk that the results would be less directly transferable to real-life situations in which the objective evaluation of products remains relevant.

In summary, our findings could provide valuable insights for the development of measures to promote/prevent the purchase of healthy/unhealthy foods—and thus combat the obesity crisis—by using descriptive norm feedback or its implementation on social media platforms (particularly considering the visual features of polls). However, for these measures to have political implications, for example, it must first be examined whether participants stated purchase intentions in our studies lead to concrete behavior (see our discussion of the intention-behavior gap in the introduction). Secondly, as also recommended by BIT (72), it must be examined whether our results can be reproduced in other environments (see also the section on study limitations). Finally, the measures must be subject to repeated review. For example, it has been shown that a successful intervention for changing behavior, namely adding a signature field at the beginning of a form (e.g., car insurance) to increase honesty, was no longer reproducible (82).

## Data Availability

The datasets presented in this article are not readily available because only aggregated data is available by request. Requests to access the datasets should be directed to LB: lars.blaeuer@zhaw.ch.

## Footnotes

1 Beverages included.

2 People with a body mass index of ≥30 kg/m^2^ are considered obese.

3 The term feedback is not consequently added.

4 Feedback should thereby preferably be based on survey data from the target group [Dempsey et al. (86); Perkins (87)].

5 However, there are also studies that found no results [see Clapp et al. (83); Granfield (84); Werch et al. (85)].

6 Note that there are also studies which combine descriptive and injunctive norms [e.g., Habib et al. (88); Schultz et al. (55)].

7 This procedure applies to all studies.

8 All studies were conducted in accordance with the ZHAW's ethical guidelines (e.g., “Z_CL_Checkliste_Ethikantraege”).

9 Note that the original language was German. You are viewing the translated versions.

10 Because unequal group sizes prevent the identification of three-way interactions (Product health × Left-side percentage × Diet).

11 A dichotomous variable was created, with purchase intention ≥50% categorized as “Yes” (the others as “No”).

12 Note, that we used a new study sample.

13 If our observation was correct, combining the data as in Study 1 should eliminate the “Product health × Left-side percentage” interaction, which was indeed the case: *F*_(1, 150)_ = 0.07, *p* = 0.788.

14 The similarities in the baseline between Studies 1 to 3 show that visual features influenced the results.

15 Some of the “Attractive” interventions overlap with those from the “Easy” domain, as simplification can also direct attention to the relevant information (72).
